# Reliability Analysis of Critical Systems in A Fuel Booster Pump Using Advanced Simulation Techniques

**DOI:** 10.3390/ma15061989

**Published:** 2022-03-08

**Authors:** Ying Luo, Yuanyuan Dong, Yuguang Li, Tian Hu, Yubei Guo, Cheng Qian, Zhihai Yang, Hao Zheng

**Affiliations:** 1 School of Chemical Engineering and Technology, Tianjin University, Tianjin 300350, China; 2 Science and Technology on Reactor System Design Technology Laboratory, Nuclear Power Institute of China, Chengdu 610213, China; sshuimus@163.com (Y.D.); liyuguang1024@163.com (Y.L.); hutiannpic@163.com (T.H.); yangzhisea@163.com (Z.Y.); josieyang11@yeah.net (H.Z.); 3 School of Reliability and Systems Engineering, Beihang University, Beijing 100191, China; zy1914109@buaa.edu.cn

**Keywords:** fuel booster pump, key component, reliability simulation, sensitivity analysis

## Abstract

The fuel booster pump is one of the most vulnerable physical assets in an operating engine due to the harsh environmental and operational conditions. However, because of its high structural complexity and extreme operational conditions, the reliability design of the fuel booster pump becomes especially difficult, particularly by means of experiments. Thus, to overcome such a problem, advanced simulation techniques have become adequate solutions for the reliability assessment and analysis of a fuel booster pump at the design stage. In this paper, by considering the effects of the uncertainties of multiple design parameters, fatigue life distributions of the four key components (which are the sealing bolt, spline shaft, graphite ring, and inducer, respectively) in a fuel booster pump were first predicted by PoF-based reliability simulations. Then, through further sensitivity analysis on each key component, the design parameters most sensitive to the component mean fatigue life were detected from a total of 25 candidate parameters. These parameters include the “nominal diameter” and “preload” for the sealing bolt, “major and minor diameters of the small spline” for the spline shaft, “inside diameter” for the graphite ring, and “fuel pressure on the blade front surface” for the inducer, respectively. These sensitivity results were found to be in good agreement with the results from the qualitative cause analysis on each key component.

## 1. Introduction

The fuel boost pump undertakes a significant function to provide a continuous and stable fuel supply under any circumstances, and is subjected to harsh environments such as high temperature, high pressure, high speed, large flow, and strong vibration, which is therefore prone to the great possibility of failure [1]. Current reliability studies on the fuel booster pump have mostly focused on the failure detection, modeling, and mitigation of its key components including the sealing bolt, spline shaft, graphite ring, inducer, etc. For instance, the main failures of the spline shaft are fretting wear load and fatigue failure [2,3,4]. Cura F et al. studied the wear damage of spline coupling and found that graphene is helpful in reducing the friction coefficient and improving the wear reliability [5] whereas for the sealing bolts, the common failure modes lead to fatigue fracture, creep failure, wear-out, and bolt looseness failure [6,7,8]. Yu Q et al. studied the low cycle fatigue life of pre-tightened bolts at high temperature and proposed a new low cycle fatigue model based on the von Mises equivalent stress–strain criterion [9]. In addition, considered as the weakest part of the mechanical sealing system, the graphite ring usually fails due to the rotating friction of the moving ring and eventually falls to pieces [10,11]. Hirani H. and Goilkar S.S. explored the friction and wear characteristics of antimony-impregnated-carbon–graphite material. It was found that reduction in the roughness of the hard flange surface will reduce the wear rate and increase the seal life [12]. Finally, the main failures of the inducer include cavitation failure, low cycle fatigue failure, corrosion, and shaft connection looseness [13,14].

It can also be seen in the literature that the functional performance, structural integrity, and reliability of fuel booster pumps are often affected by various uncertainties including material variability and model uncertainty [15,16,17,18,19,20]. Therefore, in engineering practice, component failures (e.g., fatigue failure) that exist in the fuel booster pump arise randomly in nature due to the following reasons: the material properties of these components generally show a certain variability due to stochastically distributed defects; geometrical tolerances of these components are inevitable due to the manufacturing process or design margins [21]; expert cognition in reliability varies between different experts due to their different backgrounds and experience [22]; and operational data (e.g., time between failures (TBFs) and TTRs) and types of maintenance costs (corrective and preventive maintenance) are uncertain due to different maintenance strategies [23]. Unfortunately, nowadays, these uncertainties are not carefully considered in the design process of the fuel booster pump, leading to the use of design results that are usually accompanied by a large factor of safety.

Moreover, from a design perspective, the traditional reliability design method based on fault statistics can only provide improvement solutions for the product after the failures have actually occurred, and therefore it is difficult to meet the requirements of the fast advancement of the fuel booster pump under limited time and costs. In contrast, with the PoF based simulation technology, it is possible to find potential risks in design and processing during the design stage, and take corrective measures in time to realize the collaborative design between functional performance and reliability [24]. In this paper, a PoF based reliability simulation method was developed for the reliability analysis of the key components of a fuel booster bump by considering the uncertainties of multiple parameters to find the most sensitive parameters to the mean life of the component via a sensitivity analysis based on the simulation results. This proposed reliability simulation and sensitivity analysis method is capable of providing a rapid and cost-effective reliability design guide without the use of experimental data.

The rest of this paper is organized as follows. Section 2 establishes the methodology for performing the reliability simulations and sensitive analysis on four key components (including the sealing bolt, spline shaft, graphite ring, and inducer, respectively); Section 3 provides the detailed information of the reliability simulations and a short summary of the simulation results for each component; Section 4 describes the sensitivity analysis results of each component; and finally, the concluding remarks are presented in Section 5.

## 2. Methodology

**Figure 1:**
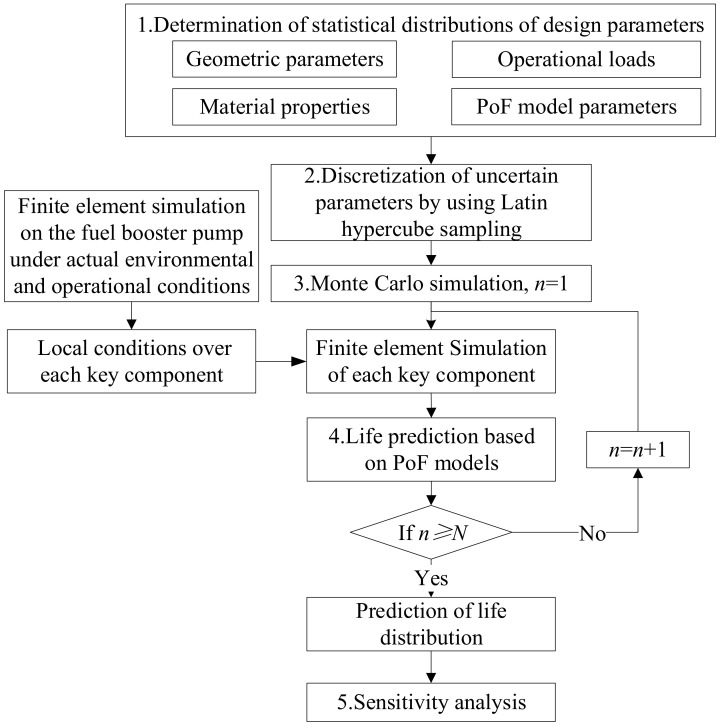
Procedure of the reliability simulation and sensitivity analysis for each key component.

The procedure to conduct the reliability simulation and sensitivity studies of each key component in a fuel booster pump is illustrated in Figure 1, which is referred to in the research [21].

1. Statistical distribution functions of all uncertain design parameters (including the geometric parameters, operational loads, material properties, PoF model parameters, etc.) of each key component are prepared first.

2. Uncertain samples for these parameters are obtained by using the Latin Hypercube Sampling (LHS) method.

3. Through the Monte Carlo simulation method, stochastic finite element simulation on each component is carried out. It is worth noting that the finite element models of the key components are automatically generated by using a parametric modeling technique. In these component simulations, it has to be noted that the local conditions (aside from the operational loads) on each component are obtained from a structural finite element simulation on the fuel booster pump under actual environmental and operational conditions.

4. The simulation results are further imported into the PoF models summarized in Table 1 for calculation of the component lives. The equations of these PoF models are provided as follows:

4(a) Basquin model

Fuel pressurization will cause components such as the sealing bolt, spline shaft, and inducer to be subjected to low amplitude alternating loads, which will eventually result in high cycle fatigue damage. Since the Basquin model expressed in Equation (1) gives good prediction performance, especially for the high cycle fatigue, it can be used in the fatigue life predictions of these components [25].

(1) $$\varepsilon_{e}=\frac{\sigma_{f}^{\prime }}{E}{\left(2N_{f}\right)}^{b},$$

where *ε_e_* indicates the elastic strain amplitude; *σ_f_^′^* indicates the fatigue strength coefficient; *b* indicates the fatigue strength index; *E* indicates the elastic modulus; and *N_f_* indicates fatigue life, respectively.

4(b) Archard wear model

Abrasive wear failure usually occurs on the contact surface between the graphite ring and moving ring. To deal with such a failure, the Archard wear model expressed in Equation (2) is used in the wear life prediction of the graphite ring [26].

(2) $$\Delta h=k\frac{p}{H}s,$$

where Δ*h* is the wear depth; *k* is the wear coefficient; *p* is the contact pressure; *s* is the relative sliding distance; *L* is the relative sliding distance; and *H* is the Brinell hardness of the material, respectively.

4(c) Fretting wear model

The study in [27] indicates that the aviation spline is usually under fretting wear contributed jointly by the abrasive wear, oxidation wear, and adhesive wear together, and one can use the fretting wear model expressed in Equation (3) to predict the wear life of the spline shaft.

(3) $$\Delta h=0.3\Delta h_{y}+0.5\Delta h_{m}+0.2\Delta h_{n}=2(0.3k_{y}+0.5k_{m}+0.2k_{n})sp,$$

where Δ*h* is the wear depth; Δ*h_y_* is the oxidation wear depth; Δ*h_m_* is the wear depth of abrasive particles; Δ*h_n_* is the adhesive wear depth; *k_y_* is the oxidation wear coefficient; *k_m_* is the abrasive wear coefficient; *k_n_* is the adhesive wear coefficient; *s* is the relative sliding distance; and *p* is the contact pressure, respectively.

In addition, assuming that the wear depth grows linearly with the load step, the wear life can be calculated by using Equation (4).

(4) $$N=\frac{h_{\max}}{\Delta h}$$

where *N* is the wear life; Δ*h* is the wear depth calculated from Equations (2) and (3); and *h_max_* is the allowable wear depth.

5. Once the relationship between the design parameters and component life prediction has been established, the sensitivity coefficients of the design parameters to the component life can be calculated by using Equation (5).

(5) $$S=\frac{\frac{N_{1}-N_{2}}{N_{1}}}{\frac{Va_{\max}-Va_{\min}}{Va_{\max}}},$$

where *S* is the sensitivity coefficient; *Va*_max_ and *Va*_min_ are the maximum and minimum values of the design parameters, respectively; *N*_1_ and *N*_2_ are the (logarithmic) life means corresponding to *Va*_max_ and *Va*_min_, respectively.

## 3. Reliability Simulation

### 3.1. LHS

The uncertain design parameters for establishing the response surfaces for life predictions of the sealing bolt, spline shaft, graphite ring and inducer are shown in Table 2, Table 3, Table 4 and Table 5, respectively. In total, there are seven parameters for the sealing bolt, and six parameters for the other three key components. The mean and coefficient of variation (CoV) values are also listed in Table 2, Table 3, Table 4 and Table 5. For each component, 250 random finite element models were generated with the design parameters in Table 2, Table 3, Table 4 and Table 5 stochastically sampled by using the LHS method. The simulation results provide the statistical distributions of the critical loading parameters (i.e., maximum von Mises strain, maximum contact stress, averages of contact stresses, etc.), which are further imported into the PoF models to calculate the component lives.

### 3.2. Finite Element Simulation

#### 3.2.1. Local Conditions of the Key Components

**Figure 2:**
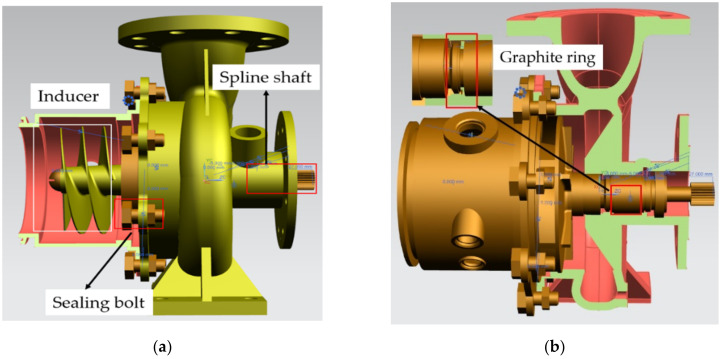
Sectional view of the fuel booster pump: (**a**) left profile; (**b**) right profile.

Among the many components in a fuel booster pump, the inducer, spline shaft, sealing bolts, and graphite ring are the most critical elements prone to failure, according to a set of studies in the literature [32,33,34,35]. Therefore, these four key components, which are illustrated in Figure 2 [36,37,38,39], were selected for reliability analysis in this study. Since they are hidden inside the fuel booster pump, the local load conditions of each component are not possible to measure experimentally with sensors, but can be obtained from a structural finite element simulation of the fuel booster pump under the actual environmental and operational conditions. These global loadings on the fuel booster pump include spline shaft torque, fuel pressure of inducer, impeller and volute, lubricating oil pressure, etc.

**Figure 3:**
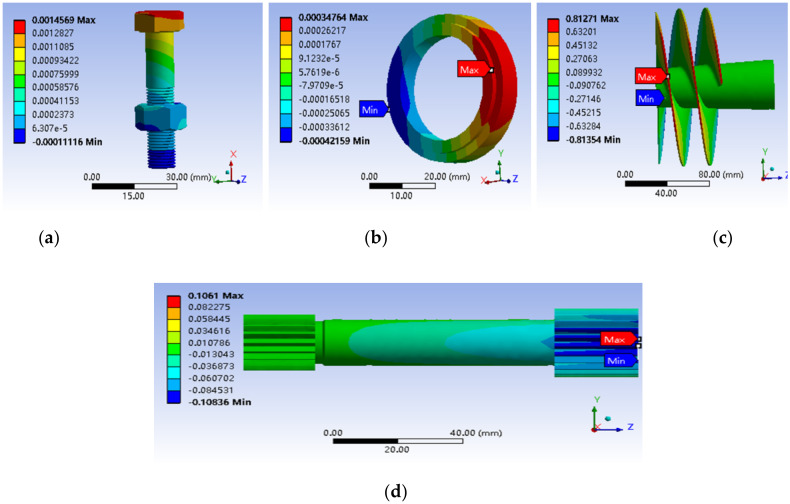
Distribution of displacement of the four key components: (**a**) sealing bolt; (**b**) graphite ring; (**c**) inducer; (**d**) spline shaft.

Through the structural finite element simulation on the fuel booster pump, displacements in the X, Y, and Z directions over the four key components can be obtained, respectively. For instance, the Y directional displacements of the four key components are shown in Figure 3a–d as examples. Furthermore, the local displacements applied on the assembly surfaces of the key components listed in Table 6 were extracted for further reliability simulations.

#### 3.2.2. Sealing Bolt

**Figure 4:**
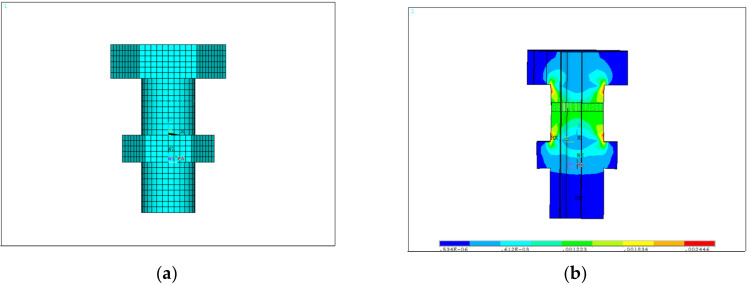
Longitudinal section of a sealing bolt model: (**a**) finite element model; (**b**) simulation results of the von Mises strains.

Eight sealing bolts are used to connect the pump casing flange and volute flange and also play a role in fixation and sealing. These sealing bolts were made of a GH4169 alloy with a density, elastic modulus, and Poisson’s ratio of 8.24 × 10^3^ kg/m^3^, 2.00 × 10^5^ MPa, and 0.32, respectively. The finite element model of the sealing bolt was established by using a parametric modeling approach with a set of geometric parameters, operational loads, material properties, and PoF model parameters. Figure 4a illustrates a random sealing bolt finite element model in which the boundary conditions have two aspects: (1) local conditions obtained from the fuel booster pump structural simulation; and (2) preload by Equation (6) applied to the cross-section of the bolt screw [40].

(6) $$F=0.7\sigma_{s}A_{s},$$

where *σ_s_* is the nominal ultimate strength of the bolt and *A_s_* is the cross-sectional area of the bolt. Figure 4b shows the simulation results of the von Mises strains in which the maximum von Mises strains of the sealing bolt are located at the interface between the screw and the nut, and used for calculating the fatigue life of the sealing bolt by using the Basquin model.

**Figure 5:**
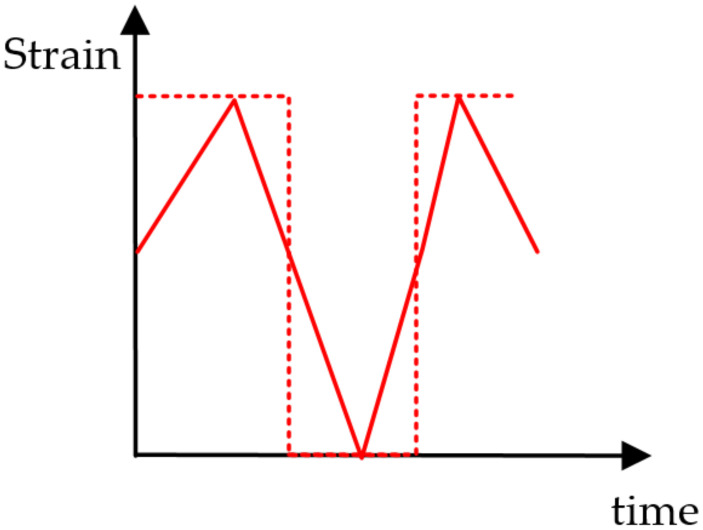
Determination of alternating loads of the sealing bolt.

In actual practice, the bolt is subjected to an alternating load illustrated by the solid line in Figure 5. However, it will require a huge computational cost to achieve a complete simulation with a full load cycle. In order to simulate the most severe condition, the maximum von Mises strain calculated above was regarded as the amplitude of the alternating elastic strain, shown in the dotted line in Figure 5, to obtain the conservative fatigue life predictions [41].

#### 3.2.3. Spline Shaft

**Figure 6:**
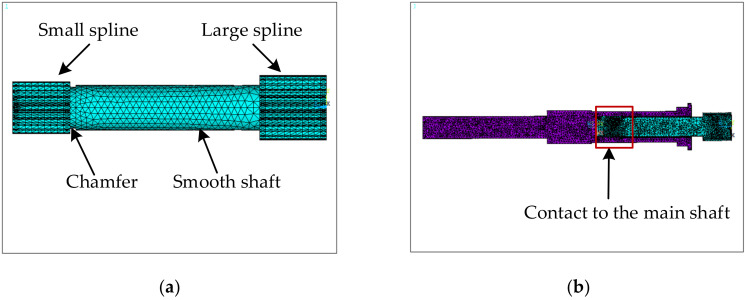
The finite element model of a spline shaft: (**a**) overall model; (**b**) an extended model with connection to the main shaft.

When the fuel booster pump works at high speed, the spline shaft is subjected to critical loadings, so it is prone to a competitive failure between wear in the spline tooth and fatigue on the shaft. The spline shaft was made of a 40CrNiMoA alloy whose density, elastic modulus and Poisson’s ratio were 7.83 × 10^3^ kg/m^3^, 2.09 × 10^5^ MPa and 0.3, respectively. Similar to the sealing bolts, the finite element model of a spline shaft was also established with design parameters related to its small spline, chamfer, smooth shaft, and large spline by using the parametric modeling approach, and illustrated in Figure 6a. In addition, Figure 6b shows an extended spline shaft model that is connected to the main shaft (made of the same material) via an internal spline. The internal spline of the main shaft is set in frictional contact with the small spline of the spline shaft, with a friction coefficient of 0.1. The boundary conditions of the spline model have two aspects: (1) local conditions obtained from the fuel booster pump structural simulation; and (2) full constraints from the main shaft.

**Figure 7:**
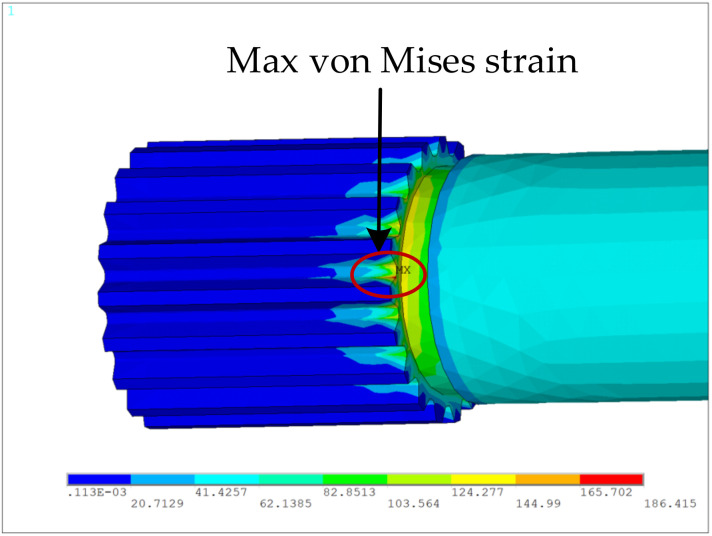
Distribution of the von Mises strains of spline shaft model.

**Figure 8:**
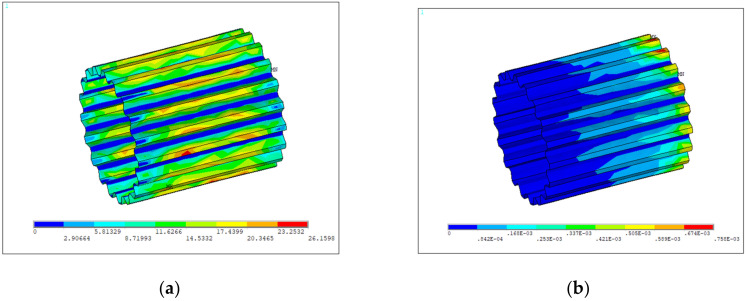
Distribution of the contact parameter results of the spline shaft model: (**a**) relative sliding distance; (**b**) contact stress.

Figure 7 shows the simulation results of von Mises strains, in which the maximum von Mises strain is located at the chamfer of the small spline, and used for calculating the fatigue life of the spline shaft by using the Basquin model. Furthermore, the sliding distances and contact stresses over the tooth of the small spline are shown in Figure 8a,b. By considering that the wear failure initiates from the most critical point, the maximum relative sliding distance and the maximum contact stress can be determined to calculate the wear life of the spline shaft using the fretting wear model. Then, according to the competitive failure principle, the failure of the spline shaft is determined by the first occurrence of either shaft fatigue or tooth wear.

#### 3.2.4. Graphite Ring

**Figure 9:**
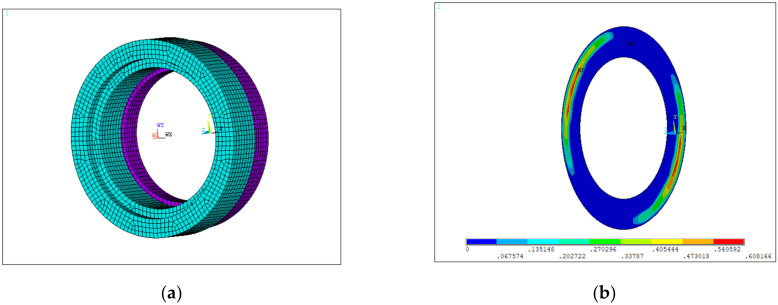
The graphite ring model in an oblique view: (**a**) finite element model; (**b**) simulation results of contact stresses.

The graphite ring is embedded in a moving ring, and is always abrasively worn against the inner surface of the moving ring. Therefore, in this study, the graphite ring model was also established including the moving ring by the parametric modeling approach. The graphite ring and moving ring were made of the M298K and 9Cr18 alloys, respectively, of which the material properties are shown in Table 7. Figure 9a shows the finite element model of the graphite ring (and the attached moving ring), in which the interface between the inner surface of the moving ring and outer surface of the graphite ring was set in frictional contact with a friction coefficient of 0.05. The boundary conditions of the graphite ring were observed from four aspects: (1) local conditions obtained from the fuel booster pump structural simulation; (2) X and Y directional constraints on the left side surface of the graphite ring; (3) X directional constraints on the right side surface of the moving ring; and (4) spring pressure of 6.5 × 10^−3^ MPa on the right side of the moving ring.

Figure 9b shows the simulation results of contact stresses, the average of which was used to calculate the wear life of the graphite ring by using the Archard model.

#### 3.2.5. Inducer

**Figure 10:**
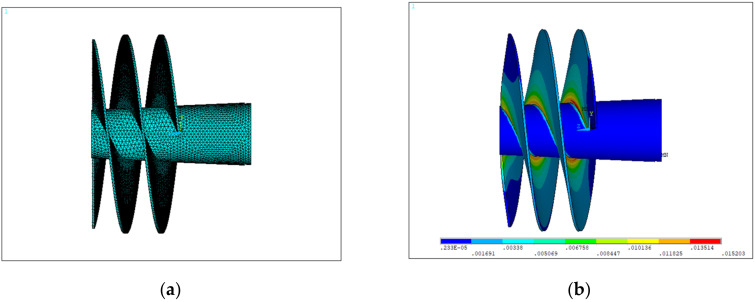
The inducer model in a front view: (**a**) finite element model; (**b**) Simulation results of von Mises strains.

In the fuel booster pump, the inducer is installed on the main shaft, and rotated driven by the rotation of the main shaft. This was made of the 2A14-T6 aluminum alloy with a density, elastic modulus, and Poisson’s ratio were 2.69 × 10^3^ kg/m^3^, 7.18 × 10^4^ MPa, and 0.33, respectively. The parametric model of the inducer is shown in Figure 10a. The boundary conditions of the graphite ring were from three aspects: (1) local conditions obtained from the fuel booster pump structural simulation; (2) Z directional constraints on the left and right ends of the inducer; and (3) fuel pressures of 0.5MPa and 0.3MPa on the front and back surfaces of the inducer, respectively.

Figure 10b shows the simulation results of the von Mises strains, in which the maximum von Mises strain was located at the root of the inducer blade, and used for calculating the fatigue life of the inducer by using the Basquin model.

## 4. Results and Discussions

### 4.1. Prediction of Life Distributions

**Figure 11:**
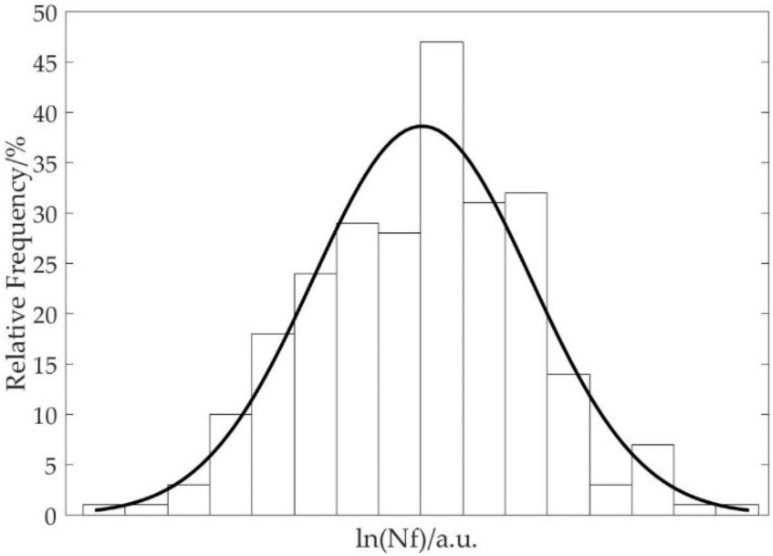
Distribution of predicted lives of the sealing bolts in logarithmic sale.

For the sealing bolt, spline shaft, and inducer, the maximum von Mises strains of these components were calculated from the corresponding stochastic finite element simulations and imported into the Basquin model as the elastic strain amplitudes for the prediction of life distribution by fatigue. Meanwhile, for the graphite ring and spline shaft, the normal loads on the contact surface were calculated from the corresponding stochastic finite element simulations and imported into the Archard model and fretting wear model for the prediction of life distribution by wear. It should be noted that the life calculation of the spline shaft was carried out from the competition between the shaft fatigue and spline tooth wear failures. For instance, it was found that from the 250 spline shaft models, 64 failed by fatigue whereas 186 failed by wear. Shown as an example, Figure 11 exhibits the distribution of the life predictions of the sealing bolts in logarithm scale, and the fitted Log-normal distribution through a Kolmogorov–Smirnov test. Likewise, via the stochastic finite element simulations on the other three key components, similar Log-normal distributions of the component lives can also be obtained.

**Figure 12:**
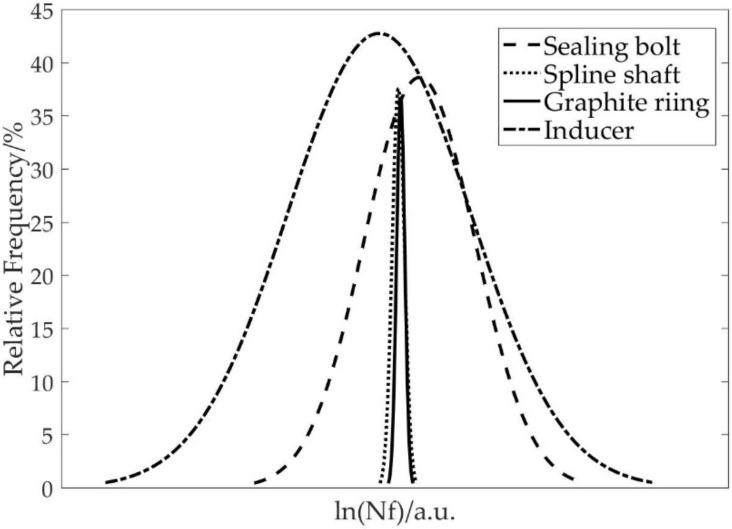
The logarithmic life fitting curve of key components.

Figure 12 shows a comparison among the predicted life distributions of the four key components in a logarithm scale. As can be seen from Figure 12, the mean lives of these four key components were relatively comparable, but the standard deviations were quite different. Obviously, the sealing bolt and inducer showed much higher dispersion degrees in the predicted lives than the spline shaft and graphite ring. It can also be seen that the characteristics (such as “mean value”) of these life distributions were altered by the integrated effects of all uncertain design parameters. However, different parameters will play different roles in changing the component life. Furthermore, one can undoubtedly design the reliability of the key components or even the fuel booster pump by considering the uncertainties of all parameters. In contrast, it is necessary to find the critical parameters with strong influence on the component life prediction through a sensitivity study, which will be discussed in detail in the following subsection.

### 4.2. Sensitivity Analysis

For each key component, sensitivity coefficients of all design parameters to the component mean life were calculated by using Equation (4), based on the reliability simulation results. For purposes of comparison, these sensitivity coefficients were ranked in Table 8, in which the positive and negative signs represent the effect played in positive and negative ways, respectively. A detailed discussion on the sensitivities of different parameters for each key component is given as follows:

(1) Sealing bolt

In the fuel booster pump, failure of the sealing bolt will cause a sealing failure between the volute and pump cover, which increases the risk of fuel leakage. From the aspect of sensitivity levels, the nominal diameter *d*, fatigue strength index *b*_1_, and preload *F* had the greatest impacts on the mean fatigue life of the sealing bolt. Among these three parameters, *b*_1_ acted as a negative exponential factor in the Basquin model. Therefore, a small increase in *b*_1_ would lead to an obvious decrease in the predicted fatigue life. The parameters *d* and *F* belong to the geometrical parameters and operational loads, respectively. They exhibited strong sensitivities to the sealing bolt fatigue life because they play significant roles in calculating von Mises strains in the finite element simulations. This agrees with the observations in the literature [36,42], which also claim that the nominal diameter and the preload have a great influence on the fatigue lives of the bolts.

In addition, the fatigue strength coefficient *σ*_f1′_, height of nut *m*, and inner circle diameter of nut *s* also exhibited moderate sensitivities to the mean fatigue life of the sealing bolt. It can also be seen that the impact of the pre-exponential factor (i.e., *σ*_*f*1′_ on the predicted fatigue lives was much less than that of exponential factor b), according to the characteristics of exponential law. *m* and *s* are both geometric parameters associated with the nuts. Small fluctuations in them will not cause great changes in the calculation of the maximum von Mises strains of sealing bolts as well as their fatigue lives.

Finally, the modulus of elasticity *E*_1_ has very limited influence on the mean fatigue life of the sealing bolt. This is because *E*_1_ influences the fatigue reliability from two completely opposite directions. On one hand, it has a negative effect on the maximum von Mises strain calculated from the finite element simulation and therefore is positive in improving the fatigue life predicted from the Basquin model. On the other hand, *E*_1_ is the pre-exponential factor in the Basquin model and plays a negative role in increasing the fatigue life. While they counteract each other, the modulus of elasticity has a very limited influence on fatigue reliability.

According to the sensitivity analysis results, a design guide for improving the sealing bolt fatigue life can be proposed by paying more careful attention to parameters such as nominal diameter *d* and preload *F*. For instance, under a certain technological level, the bolt fatigue life might be sufficiently increased by using thick bolts or by appropriately reducing the preload applied on the bolts. Nevertheless, reduction in the preload must be controlled within a reasonable level to avoid other failures such as loose bolts and wear in practice.

(2) Spline shaft

Failure of the spline shaft will affect the power transmission efficiency, thus reducing the fuel supply capacity. Most importantly, minor and major diameters *D*_2_ and *D*_1_ of the small spline had the strongest influence on the life of the spline shaft. The decrease in *D*_2_ would lead to an obvious reduction in the shaft section area, whereas the increase in *D*_1_ will increase the number of the teeth. Both actions will result in significantly higher stress/strain levels to reduce the life of the spline shaft, regardless of whether it is driven by either shaft fatigue or tooth wear. In [43], a similar argument was also drawn by claiming that the increase in the number of teeth plays an important role to enhance the strength of the spline shaft. Therefore, appropriate treatments on the parameters *D*_2_ and *D*_1_ will be the key issues to ensuring a long service life for the spline shaft.

Meanwhile, the fatigue strength coefficient *σ*_f3′_ was also sort of sensitive to the spline shaft life, but not as strong as the *D*_2_ and *D*_1_ parameters. In contrast, the elastic modulus *E*_2_ of the spline shaft, oxidation wear coefficient *k*_y_, and chamfer *R* were significantly less sensitive to the life of the spline shaft.

(3) Graphite ring

Similar to the sealing bolt, failure of the graphite ring will also reduce the sealing ability of the fuel booster pump and lead to a high risk of fuel leakage. For the graphite ring, the dominant parameter for sensitivity analysis on the life of the graphite ring is inside diameter *d_2_*. Since the graphite ring is closely attached to the moving ring, a slight increase in *d_2_* would produce a rapid increase in the contact stress between the graphite ring and the moving ring, which would significantly accelerate the abrasive wear failure. Moreover, the abrasive wear coefficient *k*_n_ and spring pressure *p* had a moderate sensitivity to the life of the graphite ring, probably in the following ways. The wear coefficient *k_n_* is related to the lubrication state of the contact surface, the hardness of the grinding material, and other material properties. The spring pressure *p* provides the driving force of wear. The sensitivities of other parameters including the Brinell hardness *H,* thickness *h_2_,* and elastic modulus *E*_3_ were much lower and could be ignored.

Therefore, it is necessary to ensure that the inside diameter *d*_2_ is well under control in the wear life design of the graphite ring. In addition, it is also recommended that the r wear coefficient *k*_n_ is reduced by controlling the lubrication, temperature, heat dissipation and viscosity of the seal, and paying attention to the selection of the spring to ensure that the spring pressure *p* is within a stable pressure range.

(4) Inducer

Failure of the inducer will cause a low fuel pressurization, which will reduce the fuel supply capacity. The fuel pressure on the front of blade *P*_1_ exhibited the strongest sensitivity to the fatigue life of the inducer due to the fact that it is the most critical parameter in the calculation of the maximum von Mises strain, as also indicated in the literature [44]. Next, the blade thickness *W*_0_, fatigue strength coefficient *σ_f_*_4*′*_, fuel pressure at the back of blade *P*_2_, and fatigue strength index *b*_4_ exhibited middle-level sensitivity to the inducer’s fatigue life. An appropriate increase in the blade thickness *W*_0_ is helpful to strengthen the blade structure of the inducer and reduce the maximum von Mises strain at the blade root. Finally, the least influential parameter is the elastic modulus *E*_4_, which barely affected the inducer fatigue life.

As a result, during the operational process of the fuel booster pump, the fuel pressure on the back of the blade should be in a reasonable bound, that is, the incoming fuel should maintain a stable and small pressure. In addition, sufficient blade thickness must be guaranteed to prevent the rupture of the inducer.

## 5. Conclusions

In this paper, a PoF based reliability simulation and sensitivity analysis method with consideration of multiple uncertainty parameters was developed for the purpose of reliability design. With the assistance of PoF models, the proposed method has the particular capacity to find the sensitivity parameters for the product’s reliability, without the use of experimental results. By applying the proposed method on four key components of the fuel booster pump, it was found that the parameters of the nominal diameter and preload for the sealing bolt, major and minor diameters of the small spline for the spline shaft, the inside diameter for the graphite ring, and fuel pressure on the blade front surface for the inducer, respectively, were the most sensitive to the mean life of each corresponding key component. These sensitivity results can be well explained by the qualitative cause analysis on each component. This implies that the proposed method can provide a chance to both speed up R&D time and save costs in the reliability analysis of component level products at the preliminary design stage.

However, despite its high cost-effective advantages, it has to be noted that the proposed method highly requires a solid understanding of the failure mechanisms to provide accurate reliability analysis results. This unfortunately limits the use of the proposed method in many practical situations. To extend its application scope, future work is recommended to strengthen studies in the accurate predictions of the complicated failures existing in the products, for instance, by developing coupled failure models or PoF-based and data-driven hybrid models.

## Figures and Tables

**Table 1:** The failure mechanism and failure physical model of the key components.

Key Components	Function	Failure	PoF Model
Sealing bolt	Tighten and seal	Fatigue	Basquin model
Spline shaft	Transmission torque	Wear	Fretting wear model
		Fatigue	Basquin model
Graphite ring	Mechanical seal	Wear	Archard wear model
Inducer	Guide and pressurize the fuel	Fatigue	Basquin model

**Table 2:** Statistical information of the design parameters of the sealing bolt.

No.	Parameter	Unit	Distribution	Mean	CoV
1	Fatigue strength coefficient *σ*_*f*1′_	MPa	Normal	1.57 × 10^3^ [21]	0.05 [21]
2	Fatigue strength index *b*_1_	/	Normal	−0.10 [21]	0.05 [21]
3	Preload *F*	N	Normal	6.04 × 10^4^ [28]	8.33 × 10^−2^ [28]
4	Nominal diameter *d*	mm	Normal	16.0 [29]	5.80 × 10^−3^ [29]
5	Inner circle diameter of nut *s*	mm	Normal	24.0 [29]	4.60 × 10^−3^ [29]
6	Height of nut *m*	mm	Normal	8.00 [29]	2.38 × 10^−2^ [29]
7	Elastic modulus *E*_1_	MPa	Normal	2.00 × 10^5^ [30]	0.05 [20]

**Table 3:** Statistical information of the design parameters of the spline shaft.

No.	Parameter	Unit	Distribution	Mean	CoV
1	Elastic modulus *E*_2_	MPa	Normal	2.09 × 10^5^ [31]	0.05 [21]
2	Chamfer *δ*	mm	Normal	0.200 [31]	0.25 [21]
3	Major diameter of small spline *D*_1_	mm	Normal	20.0 [31]	2.15 × 10^−3^
4	Minor diameter of small spline *D*_2_	mm	Normal	17.5 [13]	3.43 × 10^−3^
5	Fatigue strength coefficient *σ*_*f*2′_	MPa	Normal	2.04 × 10^3^ [31]	0.05 [21]
6	Oxidation wear coefficient *k_y_*	/	Normal	8.60 × 10^−9^ [27]	0.05 [21]

**Table 4:** Statistical information of the design parameters of the graphite ring.

Order	Parameter	Unit	Distribution	Mean	Coefficients of Variation
1	Thickness *h*_2_	mm	Normal	5.90	2.83 × 10^−3^
2	Inside diameter *d*_2_	mm	Normal	30.0	2.30 × 10^−4^
3	Elastic modulus *E*_3_	MPa	Normal	1.25 × 10^5^	0.05 [21]
4	Spring pressure *p*	MPa	Normal	0.0650	0.05 [21]
5	Brinell hardness *H*	HB	Normal	33.0	0.05 [21]
6	Wear coefficient *k*	/	Normal	3.30 × 10^−7^	0.05 [21]

**Table 5:** Statistical information of the design parameters of the inducer.

Order	Parameter	Unit	Distribution	Mean	Coefficients of Variation
1	Fuel pressure on the back of blade *P*_2_	MPa	Normal	0.30	0.05 [21]
2	Fuel pressure on the front of blade *P*_1_	MPa	Normal	0.50	0.05 [21]
3	Elastic modulus *E*_4_	Mpa	Normal	7.18 × 10^4^	0.05 [21]
4	Blade thickness *W*_0_	mm	Normal	1.60	9.36 × 10^−3^
5	Fatigue strength coefficient *σ*_*f*4′_	Mpa	Normal	1.37 × 10^3^	0.05 [21]
6	Fatigue strength index *b*_4_	/	Normal	−7.30 × 10^−2^	0.05 [21]

**Table 6:** Assembly surfaces of the four key components.

Key Components	Assembly Surfaces
Sealing bolt	Outer surface of the stud Lower surface of the bolt head Upper surface of the nut data
Spline shaft	Outer surface of the optical shaft data
Graphite ring	Inner ring surface
Inducer	Inner ring surface

**Table 7:** The material properties of the M298K and 9Cr18 alloys.

Material	Density (kg/m^3^)	Elastic Modulus (MPa)	Poisson’s Ratio
40CrNiMoA	7.83 × 10^3^	2.09 × 10^5^	0.30
9Cr18	7.70 × 10^3^	2.15 × 10^5^	0.26

**Table 8:** The results of the sensitivity analysis of the four key components.

Key Component	Order	Parameter	*S*
Sealing bolt	1	*d*	1.1148
	2	*b*_1_	−1.0689
	3	*F*	−0.7077
	4	*σ_f_*_1′_	0.5983
	5	*s*	0.2806
	6	*m*	0.1884
	7	*E*_1_	0.003
Spline shaft	1	*D*_2_	1.8717
	2	*D*_1_	−1.5694
	3	*σ_f_*_3′_	0.7242
	4	*E_2_*	−0.0683
	5	*k*_y_	−0.0566
	6	*R*	0.0054
Graphite ring	1	*d*_2_	0.6017
	2	*p*	−0.0705
	3	*k_n_*	−0.0696
	4	*H*	0.0673
	5	*h*_2_	−0.0221
	6	*E*_3_	−0.0007
Inducer	1	*P*_1_	−4.2274
	2	*W*_0_	0.7147
	3	*σ_f_*_4′_	0.46
	4	*P*_2_	0.4225
	5	*b*_4_	−0.2206
	6	*E*_4_	−0.0036

## Data Availability

Not applicable.
